# Supplementary material for: Machine learning-based prediction of rheumatoid arthritis with development of ACPA autoantibodies in the presence of non-HLA genes polymorphisms
Source: PLoS One. 2024 Mar 22;19(3):e0300717. doi: 10.1371/journal.pone.0300717 (PMC10959370; doi:10.1371/journal.pone.0300717)
Supplement: Attachment — Submitted filename: Review_responses_PLOS_ONE.pdf [file pone.0300717.s001.pdf]

Answers to the reviewers' comments to the manuscript

**Machine Learning-Based Prediction of Rheumatoid Arthritis with Development of ACPA Autoantibodies in the Presence of Non-HLA Genes Polymorphisms**

by Grzegorz Dudek et al.

## Editor's comments

Thank you for submitting your manuscript to PLOS ONE. After careful consideration, we feel that it has merit but does not fully meet PLOS ONE's publication criteria as it currently stands. Therefore, we invite you to submit a revised version of the manuscript that addresses the points raised during the review process.

Answer:

Thank you for sending me referee's comments on our manuscript. Please find attached the revised version. We tried to follow all referee's suggestions and we hope that they allow us to improve our manuscript. I am writing also on behalf of the remaining authors, who support submission of the revised form of the manuscript.

## REVIEWER 1

The authors express their sincere gratitude to Reviewer 1 for his/her insightful and constructive comments, which have significantly contributed to enhancing the quality of our paper. We have diligently addressed your feedback and made necessary amendments to our manuscript to align with your recommendations. Should there be any further inquiries or suggestions regarding our work, we warmly welcome your continued guidance and input.

1. Results are not convicing in terms of Accuracy and Sensibility and are not supported by an statistical validation with other approaches. In my opinion, is just an implementation of Matlab without any exploration of the parameters of the different algorithms.

Answer:

In mathematical models with medical use, especially in assessing the risk of the disease, the sensitivity and specificity of the model about 70% are satisfying, although not perfect. In the literature, there is no similar study to the presented study, but the criteria for diagnosing some rheumatic diseases have similar values of sensitivity and specificity. The best example is the binding criteria for the diagnosis of EULAR / ACR 2010 rheumatoid arthritis, whose sensitivity and specificity are estimated at 73.5 and 71.4%, respectively, despite that they determine the diagnostic standard of this frequent rheumatic disease (see PMID: 21292733). We have added this information into discussion.

In the new version of the manuscript, we use statistical tests to compare the methods. In Section 4.2 we added: "To rigorously examine the hypothesis that the predictions from each model have equivalent accuracy in predicting true class labels, we employ a mid-*p*-value McNemar test. This test, recommended by Dietterich [11], is particularly suitable when data

is limited, and each algorithm can only be evaluated once. The results of the test affirm that, at the 1% significance level, all models yield statistically indistinguishable results."

In new Section 4.1, we describe in detail the exploration of the ML models hiperparameters (see Answer to comment 2 below). Moreover, we have also added Section 4.2 in which we evaluate our models in terms of dealing with imbalanced data.

2. The parameters of the ML algorithms should be explored for optimization.

Answer:

To enhance clarity and detail in Section 4.1, we delve into the specifics of optimization, training, and evaluation processes. Our aim is to provide an exhaustive account of the model's hyperparameter selection, ensuring that it aligns precisely with your expectations and requirements. This section is structured to offer both a thorough understanding and practical insights into the decision-making process behind hyperparameter tuning.

**REVIEWER 2**

The authors express their sincere gratitude to Reviewer 2 for his/her insightful and constructive comments, which have significantly contributed to enhancing the quality of our paper. We have diligently addressed your feedback and made necessary amendments to our manuscript to align with your recommendations. Should there be any further inquiries or suggestions regarding our work, we warmly welcome your continued guidance and input.

1. Why is the random forest used in the last Fig 5, but not used in the preliminary experiments (Table 4)?

Answer:

The preliminary experiments employed Random Forest, a type of Boosted Trees (BT), as detailed in updated Section 4.1: "BT: Ensemble method was searched among AdaBoost, LogitBoost (adaptive logistic regression), GAB, and Random Forest." Among these, GAB exhibited superior performance and was consequently chosen for further experimentation. We return to Random Forest in the "Feature importance" section, to identify the most important features. Random Forest enables the assessment of feature importance, unlike other boosted tree algorithms, including GAM.

2. Table 3 does not list the hyperparameters for random forest.

Answer:

This is because GAB, not Random Forest, was selected as the ensemble method of BT.

3. On Page 10 (line 278), is reference 13 an appropriate citation? It seems to be a paper mentioning macrophages and autoimmune diseases.

Answer:

We have removed unnecessary paragraph as well as an appropriate citation.

4. The paragraph on Page 10 (lines 277-284) seems unnecessary.

Answer:

We have removed unnecessary paragraph.

5. On Page 10, line 287, is there significance to the sensitivity and specificity of about 70%? How would this be utilized clinically?

Answer:

We justify this in the discussion section: "In mathematical models with medical use, especially in assessing the risk of the disease, the sensitivity and specificity of the model about 70% are satisfying, although not perfect. In the literature, there is no similar study to the presented study, but the criteria for diagnosing some rheumatic diseases have similar values of sensitivity and specificity. The best example is the binding criteria for the diagnosis of EULAR / ACR 2010 rheumatoid arthritis, whose sensitivity and specificity are estimated at 73.5 and 71.4%, respectively, despite that they determine the diagnostic standard of this frequent rheumatic disease."

**REVIEWER 3**

The authors express their sincere gratitude to Reviewer 3 for his/her insightful and constructive comments, which have significantly contributed to enhancing the quality of our paper. We have diligently addressed your feedback and made necessary amendments to our manuscript to align with your recommendations. Should there be any further inquiries or suggestions regarding our work, we warmly welcome your continued guidance and input.

1. The Abstract should have the main findings communicated in it.

Answer:

The abstract has been revised to encompass the key findings of our study. We trust that it now aligns with your expectations.

2. The study settings should be clarified.

Answer: The study settings have been further elucidated in Section 5: Experimental Study. Notably, we have introduced a new subsection, 4.1, describing in detail the training, optimization, and evaluation procedures for our machine learning models. Furthermore, in subsection 4.2, we delve into an analysis of how these models address the challenge of imbalanced data.

3. Revise figure legends.

Answer:

The labels of Figs. 1 and 4 were corrected.

4. Consider the imbalance in datasets when interpreting the models.

Answer:

In new section 4.2 we discuss the problem of imbalanced data in the context of our research.

**REVIEWER 4**

The authors express their sincere gratitude to Reviewer 4 for his/her insightful and constructive comments, which have significantly contributed to enhancing the quality of our paper. We have diligently addressed your feedback and made necessary amendments to our manuscript to align with your recommendations. Should there be any further inquiries or suggestions regarding our work, we warmly welcome your continued guidance and input.

1. In the abstract, the authors should determine which type/(s) of ML model have been included. Besides, the comparison of other methodologies or even comparison with different ML models should be mentioned with some important results.

Answer:

The abstract has been updated to include information about the applied ML models and to highlight the key findings of our study. We believe it now aligns with your expectations.

2. The presentation and organization of the manuscript are poor.

Answer:

The manuscript has undergone substantial revisions, including updates to the Abstract, Introduction section, as well as the addition of two new sections: "4.1 Optimisation, Training, and Evaluation Setup" and "4.2.1 Imbalanced Data."

3. The introduction is poorly written.

Answer:

We have rewritten the introduction. We believe it is now clearer and more comprehensible to readers.

4. The authors should present their contribution clearly.

Answer:

A more comprehensive description of the contributions of this work can be found in the revised manuscript, specifically in Subsection 1.2.

5. What is the motivation of the present study?

Answer:

The aims of this study are presented in rewritten Introduction. In essence, this study aims to leverage ML to enhance the understanding and prediction of RA through genetic markers, potentially leading to more effective and individualized treatment approaches.

6. It is recommended to the author to add a descriptive paragraph at the end of the introduction illustrating the structure of the manuscript.

Answer:

The paragraph describing the structure of the manuscript was added.

7. The sections should be numbered.

Answer:

The sections are numbered.

8. The authors mentioned "Machine learning (ML) and artificial intelligence (AI) have become increasingly popular tools ...". However, ML is already under the umbrella of the AI.

Answer:

That was corrected: "Artificial Intelligence (AI), particularly Machine Learning (ML), have become increasingly popular tools …".

9. Is Genotyping an individual section or a subsection? If so, it is too short and should be merged with another section.

Answer:

"Genotyping" is a subsection of "Patient and method" section. It contains all the information necessary to repeat the experiment and check its correctness.

10. From the section called "Machine learning models". It seems the present manuscript is a comparative study. Such an important point should be clearly mentioned in the abstract and introduction.

Answer:

In the revised version, the abstract mentions that "This work is a comprehensive comparative analysis, wherein we assess and juxtapose various ML classifiers. Our evaluation encompasses a range of models, including logistic regression, $k$-nearest neighbors, naïve Bayes, decision tree, boosted trees, multilayer perceptron, and support vector machines."

Also Introduction, in Subsection 1.2, mentions that: "Evaluating ML Algorithms for RA Prediction: Various ML algorithms are assessed and compared for their effectiveness in predicting RA based on genetic data. The algorithms include logistic regression, $k$-nearest neighbors, naïve Bayes, decision trees, boosted trees, multilayer perceptrons, and support vector machines. This comparative analysis aims to determine which ML models are most effective for this purpose."

11. The authors mentioned that they use one or two classes. They should be consistent or as a minimum clarify on which criteria, they decide to use one or two.

Answer:

In our study, we use two classes: “RA” and “Healthy” (Control). We do not mention about one class.

12. The mentioned equations in page 8 have to be presented in a separate way with numbering.

Answer:

These equations are presented with numbering as (5)-(9).

13. It is normally to compare between all the model results together not to show each metric with some a group of them then change this group with other metric.

Answer:

We are not sure, we understand the Reviewer correctly. In Table 4, we compare a wide range of ML models to select the most accurate one. The selection criterion is "accuracy". In Table 5 we provide more detailed results for selected models.

14. The authors are recommended to do more surveys on the related works regarding the raised crucial comments. The following paper can assist the authors in this regard: https://doi.org/10.1109/TGRS.2022.3208097;
https://doi.org/10.1109/ACCESS.2021.3076119;
https://doi.org/10.1109/TGRS.2023.3296520

Answer:

We appreciate your insightful suggestions regarding potential applications of machine learning models. The papers you referenced, which delve into seismic intensity estimation and seismic source discrimination, indeed present intriguing approaches within their respective fields. However, the scope of our paper is distinctly focused on predicting rheumatoid arthritis (RA) using genetic data. Given this significant divergence in subject matter, we feel that citing the mentioned seismic studies in our paper might not directly contribute to its thematic coherence. Nevertheless, we acknowledge the value of interdisciplinary insights and will consider such perspectives in future research where they may be more contextually relevant.

15. The confusion matrix should a low performance of classification.

Answer:

In mathematical models with medical use, especially in assessing the risk of the disease, the accuracy, sensitivity and specificity of the model about 70% are satisfying, although not perfect. In the literature, there is no similar study to the presented study, but the criteria for diagnosing some rheumatic diseases have similar values of sensitivity and specificity. The best example is the binding criteria for the diagnosis of EULAR / ACR 2010 rheumatoid arthritis, whose sensitivity and specificity are estimated at 73.5 and 71.4%, respectively, despite that they determine the diagnostic standard of this frequent rheumatic disease (see PMID: 21292733). We have added this information into discussion.

16. How did the authors optimize the hyper-parameters.

Answer:

This is clarified in new Section 4.1 Optimisation, training and evaluation setup.
